# Autoimmune neuropsychiatric disorders manifesting with psychosis

**DOI:** 10.1172/JCI196507

**Published:** 2025-10-15

**Authors:** José Maria Cabrera-Maqueda, Jesús Planagumà, Mar Guasp, Josep Dalmau

**Affiliations:** 1 Institut d’Investigacions Biomèdiques August Pi i Sunyer (IDIBAPS), Barcelona, Spain.; 2 Service of Neurology, University Hospital Clínic of Barcelona, Barcelona, Spain.; 3 CaixaResearch Institute, Barcelona, Spain.; 4 Centre for Biomedical Network Research on Rare Diseases (CIBERER), Instituto de Salud Carlos III, Madrid, Spain.; 5 Department of Neurology, University of Pennsylvania, Philadelphia, Pennsylvania, USA.

## Abstract

The increasing recognition of a new category of encephalitides that occur in association with antibodies against neuronal surface proteins has prompted the use of terms like “autoimmune psychosis” and “autoimmune psychiatric disorders.” However, although psychosis and other psychiatric symptoms can occur in autoimmune encephalitides and systemic autoimmune diseases, evidence for a distinct psychiatric entity beyond these conditions is lacking. A particularly defining condition is anti-NMDA receptor encephalitis, which has been central to promoting concepts such as autoimmune psychosis and autoimmune psychiatric disorders. While anti-NMDA receptor encephalitis can resemble primary psychiatric conditions, certain clinical features often suggest the specific diagnosis. This Review traces the development of the autoimmune psychosis concept and examines the implications of framing it as a separate entity. We discuss leading theories of psychosis and the convergence of the NMDA receptor hypofunction/glutamate hypothesis with anti-NMDA receptor encephalitis mechanisms. The interest generated by such disorders has driven uncontrolled antibody testing in psychiatric populations, often neglecting pretest probability and favoring prevalence over diagnostic specificity. Finally, we highlight the main limitations of current approaches and propose directions for future research.

## Psychosis and its causes

A psychotic disorder refers to a set of symptoms collectively known as psychosis (Table 1), which can arise from various underlying conditions (1). Psychotic symptoms are most often associated with mental illnesses but can also result from systemic autoimmune or inflammatory diseases, neurologic disorders (e.g., genetic, neurodegenerative, brain tumors, epilepsy, encephalitis), organ failure, or metabolic, toxic, drug-related, or iatrogenic causes (2–8). Schizophrenia spectrum disorders, the prototypical group of mental illnesses causing psychosis, are manageable but not curable, with a continued risk of recurrent psychotic episodes. In contrast, many acquired causes of psychosis are both treatable and curable upon resolution of the underlying condition. While the exact causes and mechanisms of schizophrenia remain poorly understood, systemic and neurologic disorders associated with psychotic features are better characterized and often more effectively prevented. These concepts, along with observations that schizophrenia and related disorders are more prevalent in patients with inflammatory or autoimmune diseases (and vice versa), have periodically sparked interest in immune mechanisms as potential contributors to psychiatric illnesses (9–11). Evidence suggesting that treating these conditions may positively influence mental health has reinforced this interest.

This Review explores the psychotic features of autoimmune encephalitides, particularly anti-NMDA receptor (NMDAR) encephalitis, and examines whether neuronal antibody-mediated psychosis or distinct psychiatric disorders may occur independently of these conditions.

## Inflammation and autoimmunity in psychiatric disorders

The earliest evidence linking immune activation to psychiatric diseases dates back over a century, when it was proposed that infections could trigger behavioral and psychiatric symptoms (12, 13). These observations were likely related to innate immune activation affecting brain function, increasing the risk of psychosis, depression, and other mental disorders. Conversely, some studies proposed that certain infections might have therapeutic potential in psychiatric conditions, such as employing malaria inoculation to treat syphilitic psychosis (14, 15). Other researchers proposed that adaptive immunity played a role in mental diseases. For instance, in 1937, Lehman Facius reported brain-reacting antibodies in the CSF of patients with schizophrenia (16), and in 1992, a macrophage-T-lymphocyte theory of schizophrenia was introduced (17).

By the 1980s, many studies on immunity and psychiatric disorders focused on the influence of the brain on immune function (e.g., depression weakening immune responses) (18, 19). In the following decade, animal and human studies reversed this view, showing that immune activation can affect brain function (20). Animal studies identified molecular pathways linking immune activation to depressive-like behaviors (21), while clinical research showed that cytokine therapies for hepatitis (22), or elevated cytokines in pancreatic cancer (e.g., IL-6), were associated with depression and other psychiatric symptoms (23). More recently, similar links have been observed between neuropsychiatric symptoms and cytokine storms induced by chimeric antigen receptor T cell therapy (24, 25), or viral infections such as SARS CoV-2 (26, 27).

Various environmental factors, such as infections (28), childhood trauma (29), and chronic stress (30), that elevate proinflammatory cytokines (31) have been implicated in the pathophysiology and comorbidity of psychiatric disorders, including schizophrenia, bipolar disorder, major mood disorders, suicidal behavior, and posttraumatic stress disorder (32–34). Importantly, the same inflammatory pathways contribute to epilepsy, diabetes, cardiovascular disease, and osteoporosis, which often co-occur with psychiatric conditions (35). Disorders involving the adaptive immune system, such as multiple sclerosis, Guillain-Barré syndrome, systemic lupus erythematosus (SLE), celiac disease, autoimmune thyroid disorders, type 1 diabetes, and rheumatoid arthritis, have also been associated with psychotic disorders (9–11).

More recently, genome-wide association studies and gene expression profiling have identified numerous genes related to risk of schizophrenia spanning a variety of biological pathways. These include immune function (e.g., HLA-DRB1, HLA-DQA1), interleukins (e.g., IL-1A, IL-6, IL-10), synaptic plasticity and function (dopamine, glutamate, GABA, and serotonin function), ion channels (e.g., CACNA1B, CACNA1C, CACNA1H), signaling peptides (e.g., PIK3CA, PIK4CA), brain development (e.g., NRG1, RELN), and metabolism (e.g., CYP1A2, CYP2C19, CYP2D6) (36–38).

Thus, over time, diverse lines of research in schizophrenia and related psychiatric disorders have pointed to an interplay of genetic and environmental factors involving immune pathways, inflammation, synaptic function, and receptors such as NMDAR.

## NMDAR and other autoimmune encephalitides

In 2005, a report detailing four young women with a newly identified neuropsychiatric syndrome — marked by initial psychotic symptoms and the presence of autoantibodies targeting a neuronal cell surface protein, later characterized as the NMDAR — reignited the interest in autoimmune neuropsychiatric disorders (39, 40). Key features of this disease are that the autoantibodies are of the IgG class, target the GluN1 subunit of NMDAR, and are consistently present in the CSF, though they are variably present in serum, with approximately 15% of patients being seronegative (41, 42).

The estimated annual incidence of anti-NMDAR encephalitis is 1.2–2 cases per million people, varying by ethnicity, and primarily affecting children and young adults (median age ~21 years) (42–44). Approximately 70% of patients are female. Known triggers include tumors, most commonly ovarian teratomas, and less frequently herpes simplex encephalitis; however, in 50%–60% of cases, the trigger remains unidentified (42, 45). The disease often presents with isolated psychotic and behavioral symptoms, making early differentiation from primary psychiatric disorders challenging (46, 47). Although most patients develop additional neurological symptoms within days or weeks, such as seizures, abnormal movements, or reduced consciousness, 5%–10% exhibit predominantly psychotic or behavioral change throughout, often with early-onset insomnia (42, 48). The complexity of these presentations has led to efforts to define a distinct psychopathology for anti-NMDAR encephalitis (49–51) or classify them as “atypical” or “cycloid” psychosis (52, 53), though with limited clinical utility. Consequently, nearly half of patients are initially misdiagnosed with a primary psychiatric disorder, admitted to psychiatric wards, and treated with neuroleptics, increasing the risks of adverse effects that resemble neuroleptic malignant syndrome (54, 55).

Approximately 80% of patients with anti-NMDAR encephalitis experience substantial recovery; however, the disease course is prolonged (42). While functional neurological deficits and seizures tend to resolve earlier, cognitive and psychiatric symptoms can persist for many months (55–57). One study during the postacute phase found that 14% of patients transiently met criteria for schizophrenia when their recent history of encephalitis-related neurological deficits was not disclosed (55). Further analyses revealed that patients with anti-NMDAR encephalitis and those with schizophrenia exhibited similar working memory deficits in a delayed-response task. Compared with age-matched healthy participants, both populations showed a significantly reduced influence of previous stimuli on working memory content, despite memory performance being generally maintained (58). Computational simulations using a prefrontal cortex microcircuit model revealed an NMDAR-dependent process operating over longer timescales and involving mechanisms more complex than simple excitation/inhibition regulation, such as short-term potentiation (58). A key distinction was that patients with anti-NMDAR encephalitis recovered, whereas those with schizophrenia did not (55, 58), a finding supported by other studies (59).

The discovery of anti-NMDAR encephalitis was soon followed by the identification of other autoimmune encephalitides involving autoantibodies against various synaptic receptors or surface proteins (Table 2) (60). Although many of these patients present with neuropsychiatric symptoms, they are typically accompanied by concurrent or early neurological signs such as seizures, memory impairment, or altered consciousness. Abnormal findings on MRI, EEG, and CSF analysis usually help reveal the underlying neuroinflammatory process (61).

Beyond anti-NMDAR encephalitis, few studies have examined the psychotic features in other autoimmune encephalitis. One study on anti-LGI1 encephalitis — the second most common neuronal antibody-mediated encephalitis — found that, after excluding cases with neurological symptoms or those lacking core psychotic features (e.g., hallucinations, catalepsy), only 5 of 46 patients presented with isolated psychosis (62). All five patients later developed neurological deficits. A literature review in the same study identified 50 anti-LGI1 cases with reported psychosis, but only six met criteria for isolated psychosis after identical exclusions (62). Another study of 152 patients with antibodies detected by an immunoprecipitation assay — now obsolete but capable of identifying LGI1 antibodies in some cases — reported visual hallucinations in five patients and delusions in three, though only two were initially diagnosed with a primary psychiatric disorder, both with affective features (63). In the other forms of autoimmune encephalitis (Table 2), such presentations remain uncommon and largely anecdotal.

Taken together, the predominance of psychiatric symptoms, including both positive and negative psychotic features (Table 1), and the specific targeting of NMDARs uniquely distinguish anti-NMDAR encephalitis from other forms of autoimmune encephalitis.

## Mechanisms of anti-NMDAR encephalitis

**Figure 1:**
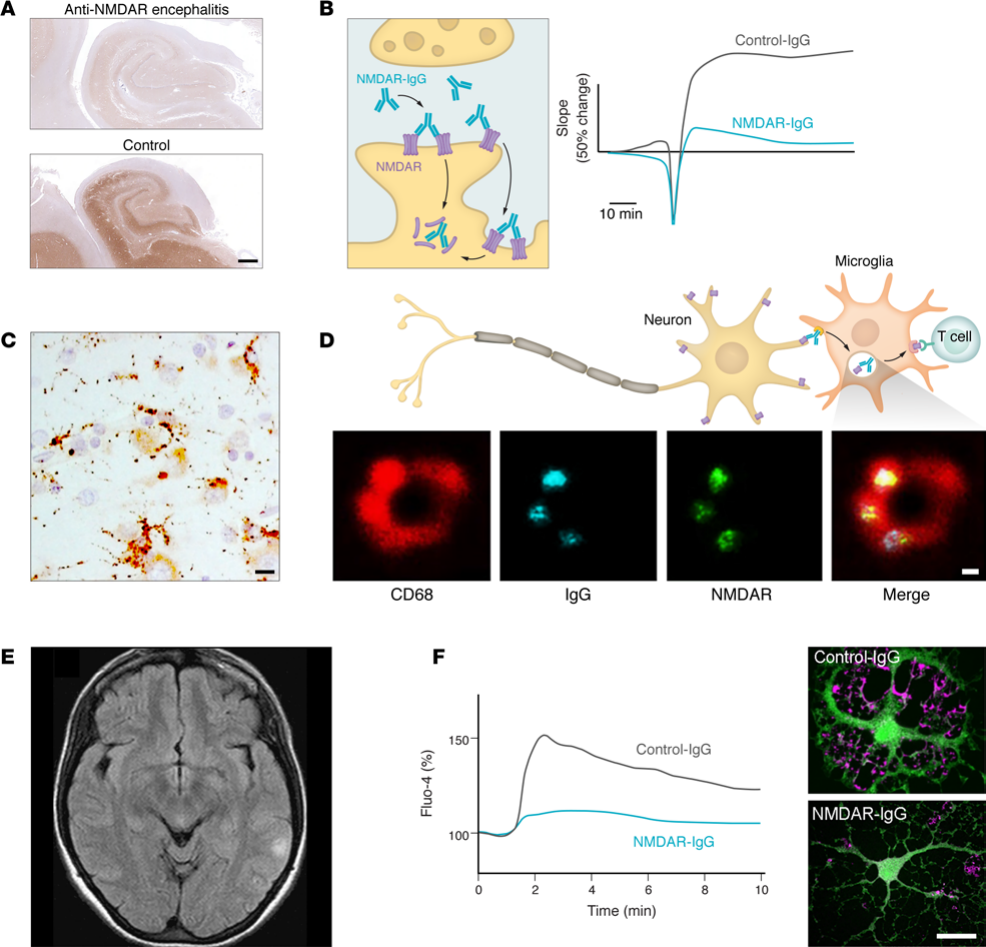
Effects of NMDAR autoantibodies on neurons and synaptic networks, microglia, and white matter. (**A**) Hippocampus from the autopsy of a patient with anti-NMDAR encephalitis (top) shows markedly reduced expression of NMDARs detected using a specific biomarker (brown immunostaining), compared with a control hippocampus (bottom) (65). (**B**) Experimental studies demonstrate that the antibodies crosslink and internalize NMDARs (left). This disrupts long-term potentiation compared with control-IgG (right) (66, 156), associated with impairment of function of NMDAR-dependent networks (72). (**C**) Microglial activation is consistently observed in patient neuropathological studies, as shown with HLA-DR staining (65). (**D**) Experimental models demonstrate that activated microglia endocytose IgG bound to NMDARs (top), with stimulated emission depletion (STED) superresolution microscopy confirming colocalization of endosomes (CD68), IgG, and the GluN1 subunit of NMDAR (bottom) (77). Microglia may process internalized NMDARs, contributing to epitope spreading and the polyclonal antibody response, likely occurring in deep cervical lymph nodes (not shown). (**E**) MRI from a patient with anti-NMDAR encephalitis shows mild increase of FLAIR signal in the left parietal region. (**F**) Although MRI studies in patients with anti-NMDAR encephalitis are often unremarkable, experimental data from cultured oligodendrocytes show that patients’ antibodies, but not control IgG, impair NMDAR-mediated calcium currents (left), which lead to reduced surface expression of GLUT1 (right; GLUT1 shown in pink), likely contributing to white matter abnormalities frequently observed with advanced neuroimaging, such as diffusion tensor imaging (81, 82). GLUT1, glucose transporter 1. Scale bars: 1 mm (**A**); 20 μm (**C**); 100 nm (**D**); 10 μm (**F**). Images were reproduced with permission from *Annals of Neurology* (65) (**A**), *Annals of Neurology* (40) (**C**), *Brain* (77) (**D**), *New England Journal of Medicine* (157) (**E**), and *Annals of Neurology* (82) (**F**).

Neuropathological studies in anti-NMDAR encephalitis reveal mild-to-moderate brain infiltrates of B cells and plasma cells that predominate over T cells, reduced NMDAR immunostaining, extensive microglial activation, and mild or absent neuronal loss (40, 64, 65). Experimental models show that patients’ antibodies exert effects at three levels: neuronal synapses and networks, microglia, and oligodendroglia and white matter (Figure 1).

Initial studies with live immunocytochemistry on cultured rat hippocampal neurons showed that NMDAR-IgG crosslinked and internalized receptors, reducing synaptic and extrasynaptic NMDAR clusters and spontaneous NMDAR-mediated currents (66). These findings associated with a disruption of the cell-surface dynamics and organization of NMDARs (67, 68). Cerebroventricular transfer of patient-derived IgG, CSF, or monoclonal antibodies to mice replicated these effects in hippocampal slices and impaired hippocampal plasticity, inducing psychosis-like behavior, anhedonia, depression-like symptoms, memory deficits, and lowered seizure threshold, all reversible upon antibody clearance (69, 70). The decrease in density of cell surface NMDARs was also associated with a decrease of dopamine 1 receptor (D1R) and increase of D2R (71). Further studies demonstrated antibody-mediated hippocampal network dysfunction, including impaired excitatory-inhibitory balance, CA1 neuronal hypoexcitability, reduced AMPA receptor signaling, and faster synaptic inhibition, ultimately triggering increased γ-oscillations in brain slices (72).

In another passive transfer model, patients’ autoantibodies were injected into the medial prefrontal cortex (mPFC) of mice, resulting in a preferential reduction in the excitability of parvalbumin (PV) neurons (73). PV neurons provide major inhibition to pyramidal neurons, and subsequent studies confirmed reduced inhibitory effects of mPFC PV neurons on local pyramidal neurons in NMDAR IgG-injected mice compared with controls. Because cortical PV neurons regulate γ-oscillations, which are important for cognitive function, the antibody-mediated reduction of NMDAR in these neurons led to a loss of NMDAR antagonist-induced cortical γ-oscillations (73). Overall, these findings resemble the effects of direct NMDAR antagonists, which are commonly used to model cognitive and psychotic features of schizophrenia (74–76).

Active immunization models, where mice generate an endogenous immune response against NMDARs, have successfully recapitulated key clinical and neuropathological features of anti-NMDAR encephalitis, including psychotic-like behavior, memory impairment, lowered seizure threshold, and abnormal movements in a subset of animals (77–80). These manifestations are accompanied by antibody-mediated reduction of synaptic NMDAR density, impaired hippocampal plasticity, and widespread microglial activation, which may contribute to epitope spreading and development of a polyclonal anti-NMDAR immune response (77).

Although standard clinical brain MRI studies are unremarkable in approximately 60% of anti-NMDAR encephalitis patients and reveal only mild-to-moderate nonspecific changes in others (42), diffusion tensor imaging studies often detect superficial white matter abnormalities that correlate with cognitive deficits (81). A potential NMDAR-specific effect on myelin integrity is supported by experiments in cultured oligodendrocytes, where patient-derived antibodies reduced NMDAR-mediated currents and decreased surface expression of the glucose transporter 1 (GLUT1), an essential component for maintaining myelin health and providing metabolic support to axons (82).

Collectively, clinical, pathological, and experimental evidence converge on a mechanism in which reduced synaptic NMDAR content and function are identified as primary drivers of the neuropsychiatric alterations in anti-NMDAR encephalitis.

## Neurotransmitter and pharmacological theories of psychosis

**Figure 2:**
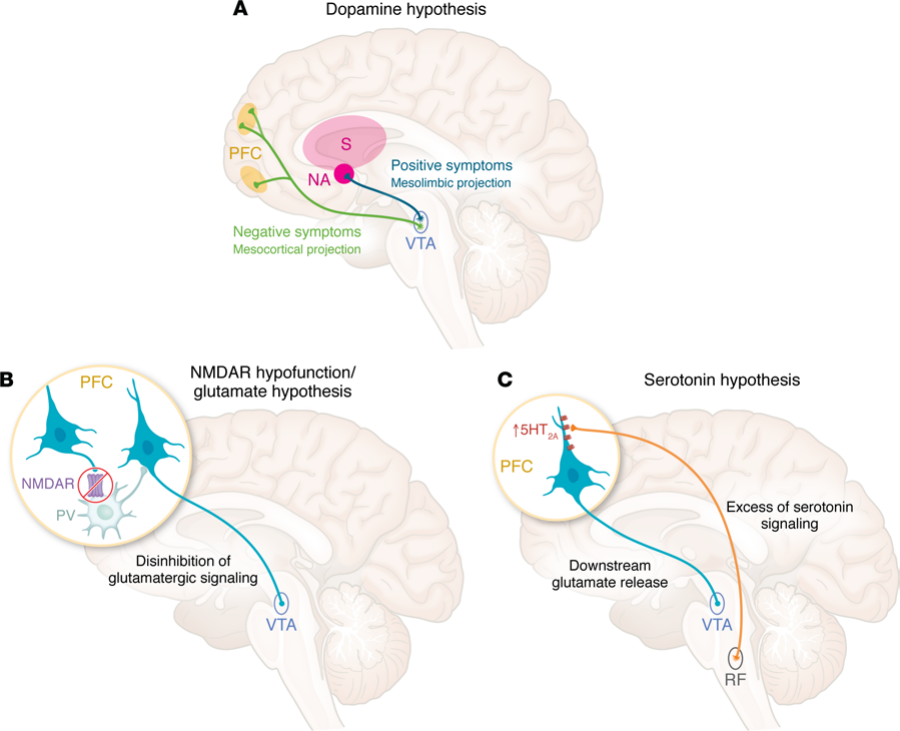
The three hypotheses of psychosis. (**A**) The classical theory of psychosis posits that dopamine hyperactivity at D2 receptors (D2Rs) in the mesolimbic pathway, particularly from ventral tegmental area (VTA) projections to the ventral striatum (nucleus accumbens), drives positive symptoms such as delusions and hallucinations in schizophrenia and manic psychosis. In contrast, dopamine hypoactivity in the mesocortical pathway is thought to underlie negative and cognitive symptoms. Typical antipsychotics that block D2Rs in the mesolimbic pathway also affect the nigrostriatal pathway (not shown), causing motor side effects such as parkinsonism and, over time, tardive dyskinesia (84). (**B**) A more recent theory of psychosis, the NMDAR hypofunction/glutamate hypothesis, proposes that dopamine hyperactivity is a downstream consequence of glutamate dysregulation in the prefrontal cortex. This hypothesis suggests that reduced NMDAR activity on GABAergic interneurons (due to neurodevelopmental abnormalities in schizophrenia or receptor internalization in anti-NMDAR encephalitis) leads to disinhibition of glutamatergic signaling, involving the VTA and activating the mesolimbic dopamine pathway. Experimental models using patient-derived autoantibodies and mouse models of anti-NMDAR encephalitis show psychosis-like behavior and reduced synaptic NMDAR content and function, supporting this mechanism (71, 73, 77). (**C**) A third theory, the serotonin hypothesis, is based on findings that 5-HT2A receptor antagonism, without D2R blockade, can be effective in treating certain forms of psychosis, such as those associated with dementia or Parkinson’s disease. In these conditions, excess serotonin signaling may result from 5-HT2A receptor upregulation, increased serotonin release, or both. This activation promotes downstream glutamate release, involving the VTA and other regions such the visual cortex, potentially contributing to symptoms like visual hallucinations (84). NA, nucleus accumbens; NMDAR, N-methyl-D-aspartate receptor; PV, parvalbumin interneuron; PFC, prefrontal cortex; RF, raphe nuclei; S, dorsal striatum; VTA, ventral tegmental area; 5-HT2A, 5-hydroxytryptamine 2A receptor.

Psychosis has traditionally been linked to a hyperdopaminergic state involving D2Rs in the ventral striatum (nucleus accumbens), part of the mesolimbic dopamine pathway (Figure 2A) (83). This theory, supported by symptom relief from D2R-blocking drugs, has been held for over 50 years. An alternative theory, the NMDAR hypofunction/glutamate hypothesis, proposes reduced NMDAR receptor function, especially in prefrontal cortical GABAergic interneurons, leading to excess glutamatergic input to the ventral tegmental area (VTA), resulting in a hyperdopaminergic state in the mesolimbic region (Figure 2B) (84, 85).

More recently, the effectiveness of pimavanserin, a 5-hydroxytryptamine 2A (5-HT__2A__, or serotonin 2A) receptor antagonist with no activity on D2R, in treating psychosis in Parkinson’s disease has suggested a third theory: the serotonin hypothesis (86). According to this model, excess serotonin release, increased 5-HT2A receptor expression, or both may trigger downstream glutamate release, which in turn stimulates the VTA (Figure 2C) (87).

Clinically, the roles of these three neurotransmitters in psychosis have long been recognized through pharmacological models. For example, it is known that psychostimulants, dissociative anesthetics, and psychedelics each induce distinct psychotic symptoms (87–90). Psychostimulants like cocaine and amphetamine increase dopamine release and D2R stimulation, typically leading to auditory hallucinations and paranoid delusions (83, 88). Anesthetics such as phencyclidine and ketamine, which are noncompetitive antagonists of NMDAR, cause visual hallucinations, paranoid delusions, and dissociative states (75, 91). Hallucinogens like LSD and psilocybin act mainly as 5-HT2A agonists, producing visual hallucinations and religious/mystical delusions (92).

Alongside the observation that different drugs can elicit varying psychotic symptoms, it is also recognized that psychosis from neurological and psychiatric disorders may present with some symptom differences. For instance, the auditory hallucinations and paranoid delusions linked to dopaminergic hyperactivity in schizophrenia differ from the visual hallucinations and persecutory or jealous delusions more commonly observed in Parkinson’s disease or dementia, where early insight is often preserved (86, 93). While D2R blockers improve the psychotic features in schizophrenia and in manic or depressive psychosis, they worsen those associated with Parkinson’s disease or dementia, which respond better to the 5-HT__2A__ antagonist pimavanserin (86).

As expected, the psychotic features of anti-NMDAR encephalitis align more closely with the NMDAR hypofunction/glutamate hypothesis than with the dopamine or serotonin models, though none provides a perfect fit (94). The clinical context and salient features for differentiating anti-NMDAR encephalitis-related psychosis are summarized in Table 3 (95). Features that frequently raise suspicion for anti-NMDAR encephalitis include the absence of a prodromal phase, the coexistence of positive and negative symptoms, pronounced fluctuations (within minutes to hours) between extreme agitation and catatonic features, manic behavior, and frequent intolerance to antipsychotic medications, particularly typical antipsychotics. Insomnia and cognitive deficits, although present in schizophrenia, tend to be disproportionately severe in the presentation of anti-NMDAR encephalitis (48, 55). Many patients show sexual disinhibition along with grandiose, religious, referential, or persecutory delusions. Hyperthermia, rigidity, decreased consciousness, elevated creatine kinase, and rhabdomyolysis, suggestive of neuroleptic malignant syndrome, may also occur in neuroleptic-naive patients (54, 96, 97).

In contrast, the biological pathways linking psychiatric symptoms to the mechanisms of other antibody-mediated encephalitides remain largely unexplored. This is due to (a) the lower prevalence of these disorders and the early overlap of neurological and psychiatric symptoms, which has led to less interest in studying the mechanisms of isolated psychosis (62, 98); (b) the absence of known autoantigens aligning with the major psychosis models, except for D2R antibody-associated encephalitis, which is exceptionally rare (98); and (c) the potential contribution of inflammation itself to psychosis, as observed in infectious (99–102) and systemic autoimmune conditions (103, 104).

Overall, clinical and mechanistic studies of anti-NMDAR encephalitis, supporting the NMDAR hypofunction/glutamate hypothesis of psychosis, along with rare psychiatric presentations in other autoimmune encephalitides, have prompted research into autoantibodies in schizophrenia and related conditions.

## Neuronal autoantibodies in schizophrenia and other psychiatric diseases

Numerous studies investigating the prevalence of autoantibodies in schizophrenia, other psychiatric disorders, and first-onset psychosis, suggest that the likelihood of identifying clinically relevant autoantibodies is low. A review of 18 series published up to 2021, collectively involving 6,573 patients with primary psychiatric disorders, found that only 50 (1%) had IgG NMDAR antibodies; a similar prevalence was observed in the control group, in which 36 (1%) of 3,893 cases had IgG NMDAR antibodies (61). In 10 of these series, no antibodies were detected in any patient with schizophrenia, schizophrenic disorders, or affective/mood disorders. Among the remaining studies, the prevalence of NMDAR antibodies in schizophrenia spectrum disorders ranged from 1% to 19%. In general, seropositive cases exhibited clinical features and outcomes comparable to those of seronegative patients. Notably, only one of the 18 studies systematically tested CSF, identifying 2 of 741 patients as “questionable” when assessing whether they were positive for NMDAR antibodies (105). Subsequent studies conducted between 2020 and 2024 have yielded similar findings (Table 4).

In the study in which 19% of patients with schizophrenia exhibited NMDAR antibodies in serum but not CSF, single-particle tracking in neurons exposed to patients’ IgG revealed disrupted NMDAR surface dynamics and receptor internalization, mirroring the effects seen in anti-NMDAR encephalitis (106). However, other studies by the same authors failed to reproduce the initially high antibody prevalence, raising doubts about missed anti-NMDAR encephalitis diagnoses in the initial series (107). A follow-up study using the same methods in CSF from patients with schizophrenia (apparently lacking NMDAR antibodies) demonstrated altered NMDAR surface dynamics and organization via unclear mechanisms (108).

It has been proposed that in psychiatric patients with serum NMDAR antibodies, only a small fraction of the antibodies crosses the blood-brain barrier, altering NMDAR surface dynamics and promoting internalization (106). The absence of detectable CSF antibodies in such cases has been attributed to their absorption by brain NMDARs (109). However, this theory remains unproven (110). Given the high antibody levels during anti-NMDAR encephalitis and their persistence at lower titers for months or years after recovery, any antibody-mediated link to schizophrenia or other psychiatric disorders would likely have been observed in individuals with prior encephalitis; however, such an association has not been observed (111, 112).

A range of autoantibodies typically associated with rheumatologic, neurologic, or paraneoplastic disorders has been reported in rare instances of schizophrenia and other psychiatric disorders. Many of these antibodies have uncertain clinical significance, even in their more common nonpsychiatric associations, such as voltage-gated potassium channels (VGKC) antibodies in limbic encephalitis (113, 114), thyroid peroxidase (TPO) in Hashimoto encephalitis (104, 105), NR2 antibodies in SLE (115), the Cunningham panel in pediatric autoimmune neuropsychiatric disorders associated with streptococcal infections (PANDAS)/pediatric acute-onset neuropsychiatric syndrome (PANS) (116), and antinuclear antibodies (ANA) in various disorders (117). The rare psychiatric cases presenting with onconeural antibodies likely reflect diagnostic inaccuracies or erroneous causal inferences, for example, the misattribution of Yo antibodies to limbic encephalitis (118). In a study of 585 patients admitted to acute psychiatric care and tested for onconeural antibodies, only one was positive for recoverin antibodies (which has no clinical value outside paraneoplastic retinopathy) (117, 119). Thus, onconeural antibody testing is not justified in well-established primary psychiatric conditions (120).

Using a different approach focused on identifying novel autoantibodies in patients with schizophrenia, researchers identified NCAM1 and neurexin-1α as targets in 5% and 2% of patients, respectively (121, 122). NCAM1 autoantibodies were also present in 1% of healthy individuals acting as controls and have been previously reported in lupus nephritis (123), whereas neurexin-1α antibodies were absent in individuals acting as controls. Both autoantibodies induced behavioral and synaptic changes in passive transfer mouse models, though their clinical significance remains unclear. The antibodies were detected a median of 24.5 and 22.5 years after schizophrenia diagnosis. Clinical features, demographics, and comorbidities were similar between antibody-positive and -negative patients, except for apparent resistance to antipsychotics in the antibody-positive group. The effect of immunotherapy was not assessed, and further studies are needed to determine clinical relevance.

Finally, some reports on schizophreniform and affective psychosis describe novel CNS autoantibodies based solely on reactivity patterns (e.g., vascular, astrocytic, granule cells, Purkinje cells, myelin tracts) (105, 124, 125). However, these studies exhibit important methodological shortcomings: antigens remained unidentified, CNS specificity was untested, controls were absent, or similar patterns were present in unrelated conditions, from obsessive-compulsive disorder to COVID-19, without clinical or therapeutic relevance. In such cases, the discovery process should match the rigor used in characterizing established autoimmune encephalitis, including the identification of specific target antigens, biological markers of CNS autoimmunity (e.g., CSF pleocytosis, elevated IgG index, autoantibodies), and well-defined syndrome-antibody associations, which may ultimately lead to the recognition of a novel autoimmune psychiatric disorder.

## Neuronal autoantibodies in first episode of psychosis

Given that psychosis is a syndrome with diverse causes, a higher detection rate of neuronal surface antibodies might be expected in first-episode psychosis compared with schizophrenia or other primary psychiatric disorders. A 2021 review of 13 studies involving 1,651 patients with first-episode psychosis found serum NMDAR antibodies in 51 cases (3%), with reported rates ranging from 0% to 12%; 8 of 11 studies reported frequencies at or below 3% (61). Among 656 individuals acting as controls, 3 (0.5%) were antibody positive. Only one study systematically tested both serum and CSF in all patients (*n =* 105), yielding negative results for NMDAR and other neuronal antibodies (126). In the remaining 12 studies, CSF was assessed in just 7 of 1,546 patients, with 4 positives — each diagnosed with anti-NMDAR encephalitis (3 with classic neurological features, 1 with isolated psychiatric symptoms).

The unexpectedly low serum autoantibody rates in first-onset psychosis raised the possibility that CSF testing might reveal more autoimmune cases (127, 128). To investigate this, a study assessed the clinical features and CSF antibodies in 105 consecutive first-onset psychosis patients (126). The median age was 30 years (range, 14–75 years), 42% of participants were female, and none had detectable neuronal antibodies. CSF pleocytosis was found in 2%, MRI abnormalities in 4%, and EEG changes in 4%, a sharp contrast to anti-NMDAR and other antibody-mediated encephalitides, where such abnormalities are detected in over 80% (CSF/MRI) and 90% (EEG) of patients (61).

Since 2021, studies examining neuronal autoantibodies in serum and CSF of patients with first episode of psychosis have reported findings consistent with earlier data (Table 5). Therefore, contrary to prior assumptions, systematic CSF testing has not identified novel psychosis-specific autoantibodies or increased rates of autoimmune causes beyond established diseases such as anti-NMDAR encephalitis.

## Implications of neuronal autoantibody findings

**Figure 3:**
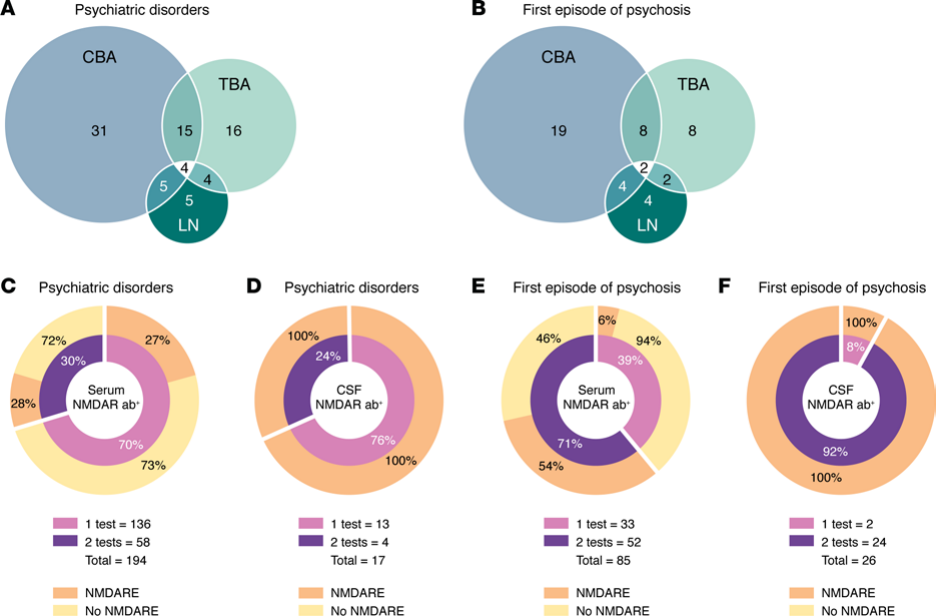
Tests and samples used in studies of neuronal autoantibodies. (**A** and **B**) Number of studies examining neuronal autoantibodies in patients with psychiatric disorders (**A**) or first episode of psychosis (**B**), categorized by the techniques used: cell-based assay (CBA), tissue-based assay (TBA), or live immunocytochemistry on cultured neurons (LN). (**C** and **D**) Number of tests (CBA and/or TBA) performed and corresponding diagnoses in patients with psychiatric disorders and NMDAR antibodies in serum (**C**) or CSF (**D**). (**E** and **F**) Number of tests (CBA and/or TBA) performed and corresponding diagnoses in patients with first episode of psychosis and NMDAR antibodies in serum (**E**) or CSF (**F**). No studies reporting NMDAR antibodies in patients with psychiatric disorders or first episode of psychosis have systematically used the three indicated techniques. Notably, all individuals with NMDAR antibodies detected in CSF, regardless of whether they were initially diagnosed with a psychiatric disorder or first episode of psychosis, were ultimately found to have anti-NMDAR encephalitis and were mostly treated with immunotherapy. In contrast, patients with NMDAR antibodies only in serum (either not tested in CSF or CSF negative), without clinical or paraclinical features of anti-NMDAR encephalitis, typically exhibited symptoms similar to antibody-negative individuals and usually received standard psychiatric care. NMDARE, anti-NMDA receptor encephalitis.

Most investigations into neuronal autoantibodies in psychiatric disorders rely on a single testing modality, typically cell-based assay (CBA; Figure 3, A and B). Among these, NMDAR antibodies are the most frequently reported, detected in 194 of 11,468 serum samples (1.7%) across 32 psychiatric cohorts published between 2010 and 2024 (Figure 3C). In CSF, NMDAR antibodies were found in 17 of 2,425 samples (0.7%) from 11 cohorts (Figure 3D).

Among patients with a first episode of psychosis, NMDAR antibodies were detected in 85 of 2,106 serum samples (4%) across 19 cohorts and in 26 of 491 CSF samples (5.3%) from 10 cohorts published between 2014 and 2024 (Figure 3, E and F).

Altogether, detection of NMDAR antibodies in CSF, whether using one or two methods, typically aligns with the clinical and paraclinical features, as well as comorbidities characteristic of anti-NMDAR encephalitis (Tables 4 and 5). In contrast, serum antibody findings are less consistent, even with identical methods, as reflected by the variability across cohorts with the same diagnoses (Tables 4 and 5) (129, 130). Similar inconsistencies are seen in control groups, with some studies reporting comparable antibody rates in healthy individuals and psychiatric patients (131–134).

Given the absent or minimal clinical differences between antibody-negative patients and those with serum-only positivity in the absence of other features of anti-NMDAR encephalitis, most experts recommend standard psychiatric care (135) and discourage neuronal antibody testing in schizophrenia and other established psychiatric disorders (136).

Similarly, in first episode of psychosis, detection of serum NMDAR antibodies without CSF positivity or other abnormalities (pleocytosis, elevated IgG index) should prompt neurology and laboratory consultation to evaluate for anti-NMDAR encephalitis and a potential false negative CSF result. In the absence of additional evidence beyond serum antibodies and isolated psychosis, immunotherapy is not indicated, and treatment should proceed with conventional antipsychotics (137).

## Current challenges and research priorities

Evidence suggests that, aside from well-characterized autoimmune encephalitides and systemic autoimmune diseases affecting the central nervous system, there is no separate autoimmune psychiatric disease, or even syndrome, beneath the broad label of “autoimmune psychosis” (117). This construct has three key limitations, each with important implications for accurate diagnosis and effective treatment.

First, the concept and criteria of “autoimmune psychosis” arose from the lack of a distinct psychiatric disease driven by autoantibodies. To be inclusive, these criteria incorporate neurological symptoms and comorbidities from multiple well-defined autoimmune or paraneoplastic encephalitides, ultimately forming a catch-all framework reliant on CSF antibody detection (138). However, when the criteria were proposed, CSF studies in reported series were scarce, and supporting evidence was virtually absent. Later studies have shown that neuronal autoantibodies are rarely tested in the CSF of psychiatric or psychosis patients, and, when tests are performed, they are typically negative, except in cases marking the onset of autoimmune encephalitides (138). Consequently, research on autoimmune psychosis has focused more on serum testing, often prioritizing prevalence over sensitivity and specificity. In practice, the criteria also exhibit limited sensitivity, missing confirmed anti-NMDAR encephalitis cases presenting with psychosis (126).

Second, psychosis can result from diverse biological pathways, each possibly requiring a different treatment approach. Some forms may respond to antipsychotic medication, while others may worsen. These differences likely apply not only to nonautoimmune psychosis, but also to the psychotic manifestations across autoimmune encephalitides. While psychotic features and other psychiatric symptoms are comparatively well characterized in anti-NMDAR encephalitis, they are largely unknown in other autoimmune encephalitides. The underlying mechanisms, whether dopaminergic, glutamatergic, or serotoninergic, also remain unknown, complicating pharmacologic management. For example, in anti-NMDAR encephalitis, where NMDAR hypofunction likely underlies psychosis, benzodiazepines are well tolerated while antipsychotics are poorly tolerated. However, the effects of these drugs on psychotic symptoms caused by other autoimmune encephalitides are unclear.

Third, clinical terminology follows a hierarchy, with well-defined diseases (e.g., anti-NMDAR encephalitis, anti-LGI1 encephalitis, SLE) representing specific diagnoses, while broader syndromes and symptoms (e.g., autoimmune psychosis) may be shared across multiple conditions and are less diagnostically specific. Reclassifying distinct diseases based on nonspecific symptoms, or grouping them by shared features such as psychosis, creates heterogeneous cohorts with differing pathophysiology, comorbidities, treatments, and outcomes. Some studies combine patients with confirmed anti-NMDAR encephalitis and those with primary psychiatric disorders and incidental, clinically ambiguous antibodies (117, 139–141). Such aggregation introduces diagnostic heterogeneity, impairs data interpretation, delays treatment, and complicates disease-specific trial enrollment.

Some of these limitations were highlighted in a study of 164 patients classified as having probable or definite autoimmune psychosis (142). Of the 119 patients with distinct neural autoantibodies, 118 had anti-NMDAR encephalitis and one had anti-LGI1 encephalitis. Three additional patients had neuropsychiatric lupus, while 42 lacked immunological characterization, leaving their specific diagnoses unclear. Unlike patients with anti-NMDAR encephalitis, the 42 unclassified cases exhibited a heterogeneous clinical profile, likely reflecting a broader spectrum of autoimmune, inflammatory, or other conditions (142). Some patients died, raising concern that established disorders were missed due to early labeling as “autoimmune psychosis without NMDAR antibodies” and limited subsequent immunological evaluation.

A clear example of the need for precise terminology and hierarchical classification in autoimmune neurology is the concept of “autoimmune epilepsy” (143). Like autoimmune psychosis, early proposals for autoimmune epilepsy included a broad set of warning signs from various autoimmune encephalitides, often misinterpreting symptomatic seizures as epilepsy (144). Later evidence highlighted the need to distinguish the underlying disease and use precise terms (symptomatic seizures vs. epilepsy) (145) given the implications for treatment decisions, social and occupational outcomes, and the fact that most autoimmune encephalitis patients do not develop epilepsy (146–148). Similar concerns arise with terms like “autoimmune obsessive-compulsive disorder,” “autoimmune depression,” “autoimmune movement disorders,” and “autoimmune dementia,” which, like autoimmune psychosis and autoimmune epilepsy, risk reframing syndromes as primary diagnoses (149, 150).

An important research priority is improving the clinical recognition of autoimmune psychiatric symptoms as manifestations of distinct autoimmune encephalitides, particularly anti-NMDAR encephalitis. This requires a focused approach, combining thorough neurological and psychiatric evaluations with serum and CSF analyses, as well as additional tests (EEG, brain MRI) to uncover the underlying encephalitis or novel autoimmune psychiatric conditions. Special attention should be given to patients with first-onset psychosis who exhibit few or no neurological symptoms but present atypically for schizophrenia, as this warrants consideration of alternative diagnoses, particularly anti-NMDAR encephalitis. These patients, as well as those who fail to respond or show intolerance to antipsychotics, regardless of the initial psychotic presentation, should undergo neurological consultation and be evaluated with EEG, brain MRI, and CSF analysis. A sequential approach, with serum antibody testing followed by CSF only if negative, is not recommended due to the risk of false positives and missed serum-negative cases with serum-only testing (111, 151).

Another priority is to avoid indiscriminate antibody testing, especially for antibodies that can occur in healthy individuals or have unclear relevance outside specific disease contexts (e.g., ANA, TPO, Zic4, recoverin, low-titer GAD, CDR2) (117, 118, 139–141, 152, 153), as they often cause diagnostic confusion, particularly when control data are lacking. Similar issues emerge when clinically relevant antibodies, like GluN1/NMDAR, are improperly tested, for example using serum in CBAs without confirmation from additional tests or CSF analysis, or when marketed diagnostics assays are suboptimal (154). These practices risk misdiagnosis and may lead to unnecessary immunotherapy in patients who would benefit more from standard psychiatric care.

In anti-NMDAR and other autoimmune encephalitides, the antibodies typically target conformational epitopes not detected by conventional ELISA, rendering results from ELISA studies of uncertain clinical significance. Given the limitations of certain antibodies and assays, tests with unclear relevance should not be used in isolation, and testing should be avoided — or results interpreted with caution — in patients with low pretest probability, as recommended for MOG-IgG in individuals meeting criteria for multiple sclerosis (155). In psychiatric patients, low pretest probability includes established chronic psychiatric illnesses or acute psychosis without neurological features or without comorbidities of autoimmune encephalitides, particularly anti-NMDAR encephalitis. Atypical features, especially those listed in Table 3, should prompt further evaluation, including CSF analysis (126).

Beyond their clinical importance, autoimmune encephalitides represent a novel category of diseases that bridge neurology and psychiatry. In these disorders, patients’ autoantibodies serve as powerful tools for investigating how immune attacks on synaptic targets can lead to psychosis and complex neuropsychiatric symptoms. Insights gained from the discovery of these disorders may prove valuable in identifying new diseases that manifest with isolated psychiatric symptoms. Active immunization models currently exist only for anti-NMDAR encephalitis, but similar approaches, paired with clinical research, could advance understanding of the neurobiology and immunobiology of related disorders (80). These models may help clarify disease mechanisms and support development of treatments beyond immunotherapy, such as allosteric modulation of targeted synaptic receptors (72, 77).

## Figures and Tables

**Table 3:**
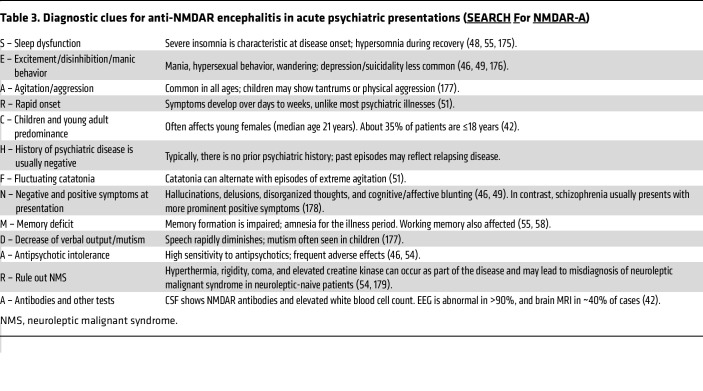
Diagnostic clues for anti-NMDAR encephalitis in acute psychiatric presentations (SEARCH For NMDAR-A)

**Table 1:**
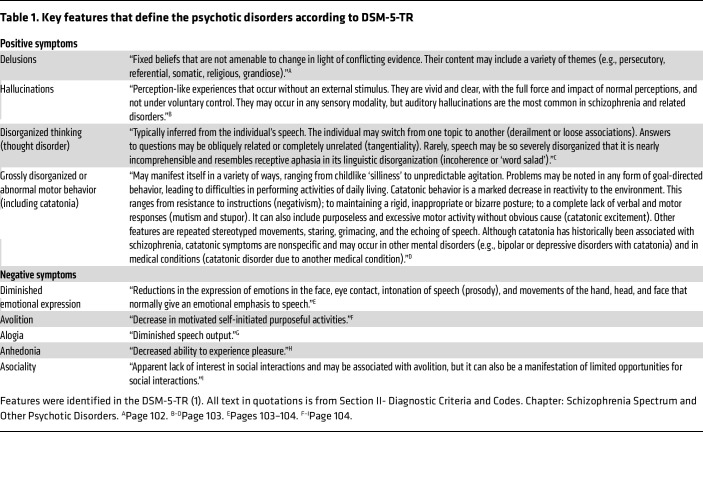
Key features that define the psychotic disorders according to DSM-5-TR

**Table 4:**
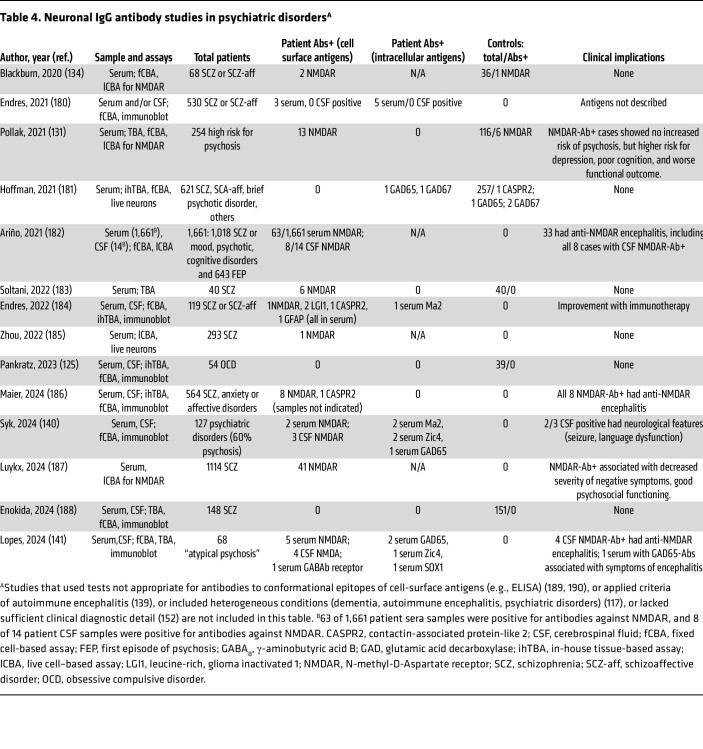
Neuronal IgG antibody studies in psychiatric disorders^A^

**Table 2:**
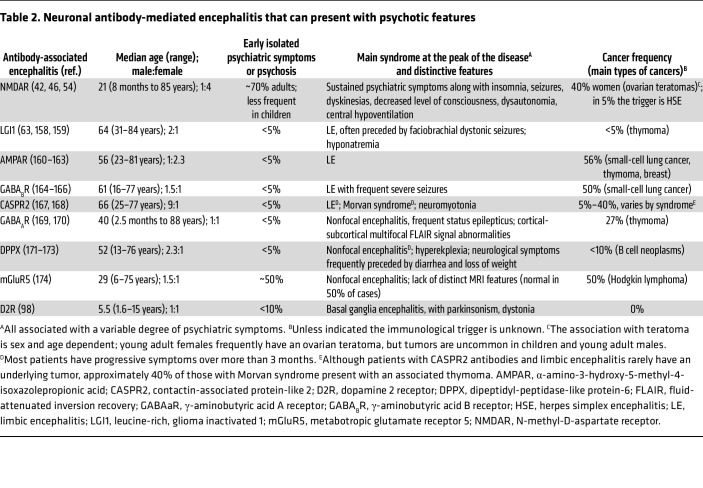
Neuronal antibody-mediated encephalitis that can present with psychotic features

**Table 5:**
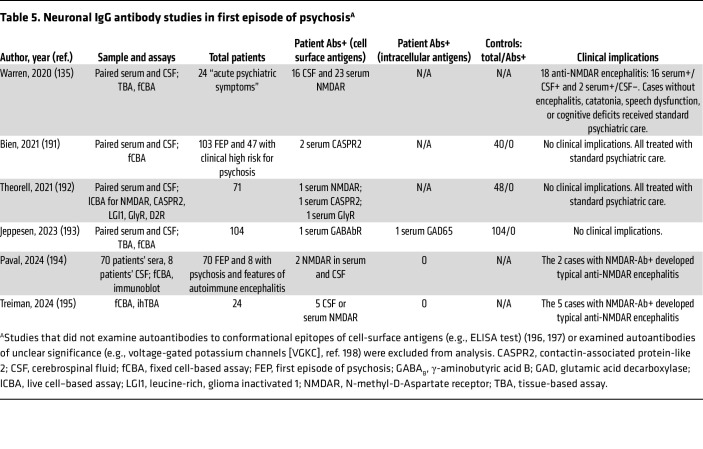
Neuronal IgG antibody studies in first episode of psychosis^A^

## Version 1 10/15/2025

Electronic publication

## References

[2] Patkar AA (2004). Psychotic symptoms in patients with medical disorders. Curr Psychiatry Rep.

[3] Isaac E (2025). Psychosis in neurocognitive disorder among ethnoculturally diverse older persons. Neurol Clin Pract.

[4] Devinsky O (2008). Postictal psychosis: common, dangerous, and treatable. Epilepsy Curr.

[5] Filley CM, Kleinschmidt-DeMasters BK (1995). Neurobehavioral presentations of brain neoplasms. West J Med.

[6] Hanly JG (2019). Psychosis in systemic lupus erythematosus: results from an international inception cohort study. Arthritis Rheumatol.

[7] Mohamed MFH (2021). Myxedema psychosis: systematic review and pooled analysis. Neuropsychiatr Dis Treat.

[8] Krystal JH (1994). Subanesthetic effects of the noncompetitive NMDA antagonist, ketamine, in humans. Psychotomimetic, perceptual, cognitive, and neuroendocrine responses. Arch Gen Psychiatry.

[9] Jeppesen R, Benros ME (2019). Autoimmune diseases and psychotic disorders. Front Psychiatry.

[10] Benros ME (2011). Autoimmune diseases and severe infections as risk factors for schizophrenia: a 30-year population-based register study. Am J Psychiatry.

[11] Benros ME (2014). A nationwide study on the risk of autoimmune diseases in individuals with a personal or a family history of schizophrenia and related psychosis. Am J Psychiatry.

[12] Menninger KA (1994). Influenza and schizophrenia. An analysis of post-influenzal “dementia precox,” as of 1918, and five years later further studies of the psychiatric aspects of influenza. 1926. Am J Psychiatry.

[13] Kamman GR (1930). Schizophrenic reactions following influenza. JAMA.

[14] Ironside RN (1925). On the treatment of general paralysis by malaria inoculation. Br J Vener Dis.

[15] Lecture TM (1929). Treatment of mental disease. Br Med J.

[16] Lehmann-Facius H (1937). Über die Liquordiagnose der Schizophrenien. Klinische Wochenschrift.

[17] Smith RS (1992). A comprehensive macrophage-T-lymphocyte theory of schizophrenia. Med Hypotheses.

[18] Kronfol Z (1983). Impaired lymphocyte function in depressive illness. Life Sci.

[19] Maes M (1989). Impaired lymphocyte stimulation by mitogens in severely depressed patients. A complex interface with HPA-axis hyperfunction, noradrenergic activity and the ageing process. Br J Psychiatry.

[20] Pariante CM (2015). Psychoneuroimmunology or immunopsychiatry?. Lancet Psychiatry.

[21] Yirmiya R (1996). Endotoxin produces a depressive-like episode in rats. Brain Res.

[22] Udina M (2016). Glucocorticoid receptors, brain-derived neurotrophic factor, serotonin and dopamine neurotransmission are associated with interferon-induced depression. Int J Neuropsychopharmacol.

[23] Breitbart W (2014). Depression, cytokines, and pancreatic cancer. Psychooncology.

[24] Pawar DS (2019). Toxicities of CAR T-cell therapy and the role of the consultation-liaison psychiatrist. Psychosomatics.

[25] Taylor MR (2023). Biobehavioral implications of chimeric antigen receptor t-cell therapy: current state and future directions. Transplant Cell Ther.

[26] Nawa H, Murakami M (2025). Neurobiology of COVID-19-associated psychosis/schizophrenia: implication of epidermal growth factor receptor signaling. Neuropsychopharmacol Rep.

[27] Dey R, Bishayi B (2023). Microglial inflammatory responses to SARS-CoV-2 infection: a comprehensive review. Cell Mol Neurobiol.

[28] Brown AS (2015). The kraepelinian dichotomy from the perspective of prenatal infectious and immunologic insults. Schizophr Bull.

[29] Danese A (2007). Childhood maltreatment predicts adult inflammation in a life-course study. Proc Natl Acad Sci U S A.

[30] Copeland WE (2014). Childhood bullying involvement predicts low-grade systemic inflammation into adulthood. Proc Natl Acad Sci U S A.

[31] Miller BJ (2011). Meta-analysis of cytokine alterations in schizophrenia: clinical status and antipsychotic effects. Biol Psychiatry.

[32] Girgis RR (2014). The cytokine model of schizophrenia: emerging therapeutic strategies. Biol Psychiatry.

[33] Black C, Miller BJ (2015). Meta-analysis of cytokines and chemokines in suicidality: distinguishing suicidal versus nonsuicidal patients. Biol Psychiatry.

[34] Berk M (2013). So depression is an inflammatory disease, but where does the inflammation come from?. BMC Med.

[35] Rosenblat JD, McIntyre RS (2015). Are medical comorbid conditions of bipolar disorder due to immune dysfunction?. Acta Psychiatr Scand.

[36] Sundararajan T (2018). Functional analysis of schizophrenia genes using GeneAnalytics program and integrated databases. Gene.

[37] Fromer M (2014). De novo mutations in schizophrenia implicate synaptic networks. Nature.

[38] Butler MG (2016). Currently recognized genes for schizophrenia: High-resolution chromosome ideogram representation. Am J Med Genet B Neuropsychiatr Genet.

[39] Vitaliani R (2005). Paraneoplastic encephalitis, psychiatric symptoms, and hypoventilation in ovarian teratoma. Ann Neurol.

[40] Dalmau J (2007). Paraneoplastic anti-N-methyl-D-aspartate receptor encephalitis associated with ovarian teratoma. Ann Neurol.

[41] Dalmau J (2008). Anti-NMDA-receptor encephalitis: case series and analysis of the effects of antibodies. Lancet Neurol.

[42] Titulaer MJ (2013). Treatment and prognostic factors for long-term outcome in patients with anti-NMDA receptor encephalitis: an observational cohort study. Lancet Neurol.

[43] Kerstens J (2024). Autoimmune encephalitis and paraneoplastic neurologic syndromes: a nationwide study on epidemiology and antibody testing performance. Neurol Neuroimmunol Neuroinflamm.

[44] Alsalek S (2024). Racial and ethnic disparities in the incidence of anti-NMDA receptor encephalitis. Neurol Neuroimmunol Neuroinflamm.

[45] Armangue T (2018). Frequency, symptoms, risk factors, and outcomes of autoimmune encephalitis after herpes simplex encephalitis: a prospective observational study and retrospective analysis. Lancet Neurol.

[46] Kayser MS (2013). Frequency and characteristics of isolated psychiatric episodes in anti–N-methyl-d-aspartate receptor encephalitis. JAMA Neurol.

[47] Kayser MS, Dalmau J (2016). Anti-NMDA receptor encephalitis, autoimmunity, and psychosis. Schizophr Res.

[48] Arino H (2020). Sleep disorders in anti-NMDAR encephalitis. Neurology.

[49] Al-Diwani A (2019). The psychopathology of NMDAR-antibody encephalitis in adults: a systematic review and phenotypic analysis of individual patient data. Lancet Psychiatry.

[50] Sarkis RA (2019). Anti-N-methyl-D-aspartate receptor encephalitis: A review of psychiatric phenotypes and management considerations: A report of the American Neuropsychiatric Association Committee on Research. J Neuropsychiatry Clin Neurosci.

[51] Warren N (2018). Refining the psychiatric syndrome of anti-N-methyl-d-aspartate receptor encephalitis. Acta Psychiatr Scand.

[52] Hinotsu K (2022). The validity of atypical psychosis diagnostic criteria to detect anti-NMDA receptor encephalitis with psychiatric symptoms. Schizophr Res.

[53] Gine Serven E (2021). Cycloid psychosis as a psychiatric expression of anti-NMDAR encephalitis. A systematic review of case reports accomplished with the authors’ cooperation. Brain Behav.

[54] Lejuste F (2016). Neuroleptic intolerance in patients with anti-NMDAR encephalitis. Neurol Neuroimmunol Neuroinflamm.

[55] Guasp M (2022). Clinical characterisation of patients in the post-acute stage of anti-NMDA receptor encephalitis: a prospective cohort study and comparison with patients with schizophrenia spectrum disorders. Lancet Neurol.

[56] Heine J (2021). Long-term cognitive outcome in anti-N-Methyl-D-aspartate receptor encephalitis. Ann Neurol.

[57] Chen LW (2024). Very long-term functional outcomes and dependency in children with anti-NMDA receptor encephalitis. Neurol Neuroimmunol Neuroinflamm.

[58] Stein H (2020). Reduced serial dependence suggests deficits in synaptic potentiation in anti-NMDAR encephalitis and schizophrenia. Nat Commun.

[59] Kelleher E (2025). Cognitive outcomes and performance of patients diagnosed and treated for N-Methyl-D-Aspartate receptor antibody-mediated (NMDAR) encephalitis compared with patients with schizophrenia and healthy controls. Psychiatry Res Neuroimaging.

[60] Dalmau J (2017). Autoantibodies to synaptic receptors and neuronal cell surface proteins in autoimmune diseases of the central nervous system. Physiol Rev.

[62] Yi Y (2025). Identifying anti-LGI-1 encephalitis in psychotic disorders: A clinically focused review. Gen Hosp Psychiatry.

[63] Somers KJ (2011). Psychiatric manifestations of voltage-gated potassium-channel complex autoimmunity. J Neuropsychiatry Clin Neurosci.

[64] Martinez-Hernandez E (2011). Analysis of complement and plasma cells in the brain of patients with anti-NMDAR encephalitis. Neurology.

[65] Zrzavy T (2021). Neuropathological variability within a spectrum of NMDAR-encephalitis. Ann Neurol.

[66] Hughes EG (2010). Cellular and synaptic mechanisms of anti-NMDA receptor encephalitis. J Neurosci.

[67] Mikasova L P (2012). Disrupted surface cross-talk between NMDA and Ephrin-B2 receptors in anti-NMDA encephalitis. Brain.

[68] Ladepeche L (2018). NMDA receptor autoantibodies in autoimmune encephalitis cause a subunit-specific nanoscale redistribution of NMDA receptors. Cell Rep.

[69] Planaguma J (2015). Human N-methyl D-aspartate receptor antibodies alter memory and behaviour in mice. Brain.

[70] Wright S (2015). Epileptogenic effects of NMDAR antibodies in a passive transfer mouse model. Brain.

[71] Carceles-Cordon M (2020). NMDAR antibodies alter dopamine receptors and cause psychotic behavior in mice. Ann Neurol.

[72] Ceanga M (2023). Human NMDAR autoantibodies disrupt excitatory-inhibitory balance, leading to hippocampal network hypersynchrony. Cell Rep.

[73] Feng YF (2025). Parvalbumin neurons mediate neurological phenotypes of anti-NMDAR encephalitis. Brain.

[74] Gonzalez-Burgos G, Lewis DA (2012). NMDA receptor hypofunction, parvalbumin-positive neurons, and cortical gamma oscillations in schizophrenia. Schizophr Bull.

[75] Javitt DC, Zukin SR (1991). Recent advances in the phencyclidine model of schizophrenia. Am J Psychiatry.

[76] Li Q (2002). NMDA receptor antagonists disinhibit rat posterior cingulate and retrosplenial cortices: a potential mechanism of neurotoxicity. J Neurosci.

[77] Maudes E (2025). Neuro-immunobiology and treatment assessment in a mouse model of anti-NMDAR encephalitis. Brain.

[78] Jones BE (2019). Autoimmune receptor encephalitis in mice induced by active immunization with conformationally stabilized holoreceptors. Sci Transl Med.

[79] Ding Y (2021). Anti-NMDAR encephalitis induced in mice by active immunization with a peptide from the amino-terminal domain of the GluN1 subunit. J Neuroinflammation.

[80] Maudes E (2025). Animal models of autoimmune encephalitis. Curr Opin Immunol.

[81] Phillips OR (2018). Superficial white matter damage in anti-NMDA receptor encephalitis. J Neurol Neurosurg Psychiatry.

[82] Matute C (2020). N-Methyl-D-aspartate receptor antibodies in autoimmune encephalopathy alter oligodendrocyte function. Ann Neurol.

[83] Meltzer HY, Stahl SM (1976). The dopamine hypothesis of schizophrenia: a review. Schizophr Bull.

[84] Stahl SM (2018). Beyond the dopamine hypothesis of schizophrenia to three neural networks of psychosis: dopamine, serotonin, and glutamate. CNS Spectr.

[85] Farber NB (2003). The NMDA receptor hypofunction model of psychosis. Ann N Y Acad Sci.

[86] Stahl SM (2016). Parkinson’s disease psychosis as a serotonin-dopamine imbalance syndrome. CNS Spectr.

[87] Vollenweider FX (1998). Psilocybin induces schizophrenia-like psychosis in humans via a serotonin-2 agonist action. Neuroreport.

[88] Mahoney JJ (2008). Presence and persistence of psychotic symptoms in cocaine- versus methamphetamine-dependent participants. Am J Addict.

[89] Weiner AL (2000). Ketamine abusers presenting to the emergency department: a case series. J Emerg Med.

[90] Carhart-Harris RL (2016). Neural correlates of the LSD experience revealed by multimodal neuroimaging. Proc Natl Acad Sci U S A.

[91] Powers AR (2015). Ketamine-induced hallucinations. Psychopathology.

[92] Griffiths R (2008). Mystical-type experiences occasioned by psilocybin mediate the attribution of personal meaning and spiritual significance 14 months later. J Psychopharmacol.

[93] Ravina B (2007). Diagnostic criteria for psychosis in Parkinson’s disease: report of an NINDS, NIMH work group. Mov Disord.

[94] Dalmau J (2016). Cotzias Lecture: NMDA receptor encephalitis and other antibody-mediated disorders of the synapse. Neurology.

[95] Dalmau J (2019). An update on anti-NMDA receptor encephalitis for neurologists and psychiatrists: mechanisms and models. Lancet Neurol.

[96] Sansing LH (2007). A patient with encephalitis associated with NMDA receptor antibodies. Nat Clin Pract Neurol.

[97] Wang HY (2019). Anti-N-methyl-D-aspartate receptor encephalitis mimics neuroleptic malignant syndrome: case report and literature review. Neuropsychiatr Dis Treat.

[98] Dale RC (2012). Antibodies to surface dopamine-2 receptor in autoimmune movement and psychiatric disorders. Brain.

[99] Bean P (2025). Psychiatric manifestations of encephalitis. Ann Clin Transl Neurol.

[100] Kulaga SS, Miller CWT (2021). Viral respiratory infections and psychosis: A review of the literature and the implications of COVID-19. Neurosci Biobehav Rev.

[101] Obuaya CC (2021). *Brucella*-induced acute psychosis: a novel cause of acute psychosis. Case Rep Infect Dis.

[102] Jarocki A (2024). Neurosyphilis-induced psychosis in Europe: a systematic review of case reports. Pathogens.

[103] Samadder A (2025). Lupus psychosis: an unusual cause of acute confusion presenting to acute medicine. BMJ Case Rep.

[104] Mattozzi S (2020). Hashimoto encephalopathy in the 21st century. Neurology.

[105] Endres D (2020). Cerebrospinal fluid, antineuronal autoantibody, EEG, and MRI findings from 992 patients with schizophreniform and affective psychosis. Transl Psychiatry.

[106] Jezequel J (2017). Dynamic disorganization of synaptic NMDA receptors triggered by autoantibodies from psychotic patients. Nat Commun.

[107] Jezequel J (2017). Cell- and single molecule-based methods to detect anti-N-Methyl-D-aspartate receptor autoantibodies in patients with first-episode psychosis from the OPTiMiSE Project. Biol Psychiatry.

[108] Espana A (2021). Alteration of NMDA receptor trafficking as a cellular hallmark of psychosis. Transl Psychiatry.

[109] Castillo-Gomez E (2016). The brain as immunoprecipitator of serum autoantibodies against N-Methyl-D-aspartate receptor subunit NR1. Ann Neurol.

[110] Dalmau J (2020). Letter by Dalmau regarding article, “Serum Anti-NMDA (N-Methyl-D-Aspartate)-receptor antibodies and long-term clinical outcome after stroke (PROSCIS-B)”. Stroke.

[111] Gresa-Arribas N (2014). Antibody titres at diagnosis and during follow-up of anti-NMDA receptor encephalitis: a retrospective study. Lancet Neurol.

[112] Hansen HC (2013). Persistent intrathecal antibody synthesis 15 years after recovering from anti-N-methyl-D-aspartate receptor encephalitis. JAMA Neurol.

[113] van Sonderen A (2017). The value of LGI1, Caspr2 and voltage-gated potassium channel antibodies in encephalitis. Nat Rev Neurol.

[114] Michael S (2020). Stop testing for autoantibodies to the VGKC-complex: only request LGI1 and CASPR2. Pract Neurol.

[115] Hanly JG (2011). Autoantibodies as biomarkers for the prediction of neuropsychiatric events in systemic lupus erythematosus. Ann Rheum Dis.

[116] Board of Directors (2025). Pediatric acute-onset neuropsychiatric syndrome (PANS): Clinical Report. Pediatrics.

[117] Hansen N (2023). Long-term course of neural autoantibody-associated psychiatric disorders: retrospective data from a specifically immunopsychiatric outpatient clinic. Antibodies (Basel).

[118] Tebartz van Elst L The neuropsychiatric checklist for autoimmune psychosis: a narrative review. Biol Psychiatry.

[119] Saether SG (2017). Onconeural antibodies in acute psychiatric inpatient care. J Neuropsychiatry Clin Neurosci.

[120] Saether SG (2017). What is the significance of onconeural antibodies for psychiatric symptomatology? A systematic review. BMC Psychiatry.

[121] Shiwaku H (2022). Autoantibodies against NCAM1 from patients with schizophrenia cause schizophrenia-related behavior and changes in synapses in mice. Cell Rep Med.

[122] Shiwaku H (2023). Analyzing schizophrenia-related phenotypes in mice caused by autoantibodies against NRXN1α in schizophrenia. Brain Behav Immun.

[123] Xia X (2024). Clinicopathological phenotype and outcomes of NCAM-1+ membranous lupus nephritis. Nephrol Dial Transplant.

[124] Franke C (2021). High frequency of cerebrospinal fluid autoantibodies in COVID-19 patients with neurological symptoms. Brain Behav Immun.

[125] Pankratz B (2023). Cerebrospinal fluid findings in adult patients with obsessive-compulsive disorder: A retrospective analysis of 54 samples. World J Biol Psychiatry.

[126] Guasp M (2021). Clinical, neuroimmunologic, and CSF investigations in first episode psychosis. Neurology.

[127] Mantere O (2018). Anti-neuronal anti-bodies in patients with early psychosis. Schizophr Res.

[128] Gaughran F (2018). Brain-relevant antibodies in first-episode psychosis: a matched case-control study. Psychol Med.

[129] Dalmau J, Graus F (2023). Autoimmune encephalitis-misdiagnosis, misconceptions, and how to avoid them. JAMA Neurol.

[130] Flanagan EP (2023). Autoimmune encephalitis misdiagnosis in adults. JAMA Neurol.

[131] Pollak TA (2021). Clinical, cognitive and neuroanatomical associations of serum NMDAR autoantibodies in people at clinical high risk for psychosis. Mol Psychiatry.

[132] Hammer C (2014). Neuropsychiatric disease relevance of circulating anti-NMDA receptor autoantibodies depends on blood-brain barrier integrity. Mol Psychiatry.

[133] Dahm L (2014). Seroprevalence of autoantibodies against brain antigens in health and disease. Ann Neurol.

[134] Blackburn KM (2020). NMDA receptor antibody seropositivity in psychosis: A pilot study from the Bipolar-Schizophrenia Network for Intermediate Phenotypes (B-SNIP). Schizophr Res.

[135] Warren N (2020). Serum and CSF Anti-NMDAR antibody testing in psychiatry. J Neuropsychiatry Clin Neurosci.

[136] Scott JG (2018). Testing for antibodies to N-methyl-d-aspartate receptor and other neuronal cell surface antigens in patients with early psychosis. Aust N Z J Psychiatry.

[137] Pollak TA (2021). Relationship between serum NMDA receptor antibodies and response to antipsychotic treatment in first-episode psychosis. Biol Psychiatry.

[138] Pollak TA (2020). Autoimmune psychosis: an international consensus on an approach to the diagnosis and management of psychosis of suspected autoimmune origin. Lancet Psychiatry.

[139] Endres D (2022). Clinical manifestations and immunomodulatory treatment experiences in psychiatric patients with suspected autoimmune encephalitis: a case series of 91 patients from Germany. Mol Psychiatry.

[140] Syk M (2024). An exploratory study of the damage markers NfL, GFAP, and t-Tau, in cerebrospinal fluid and other findings from a patient cohort enriched for suspected autoimmune psychiatric disease. Transl Psychiatry.

[141] Lopes J (2024). Blood and CSF anti-neuronal antibodies testing in psychotic syndromes: a retrospective analysis from a tertiary psychiatric hospital. Immunol Res.

[142] Ramirez-Bermudez J (2025). Autoimmune psychosis: Psychopathological patterns and outcome after immunotherapy. Schizophr Res.

[143] Toledano M, Pittock SJ (2015). Autoimmune epilepsy. Semin Neurol.

[144] Dubey D (2017). Predictive models in the diagnosis and treatment of autoimmune epilepsy. Epilepsia.

[145] Geis C (2019). Autoimmune seizures and epilepsy. J Clin Invest.

[146] Steriade C (2020). Acute symptomatic seizures secondary to autoimmune encephalitis and autoimmune-associated epilepsy: Conceptual definitions. Epilepsia.

[147] de Bruijn M (2019). Evaluation of seizure treatment in anti-LGI1, anti-NMDAR, and anti-GABABR encephalitis. Neurology.

[148] Rada A (2024). Risk of seizure recurrence due to autoimmune encephalitis with NMDAR, LGI1, CASPR2, and GABA_B_R antibodies: implications for return to driving. Neurol Neuroimmunol Neuroinflamm.

[149] Endres D (2022). Immunological causes of obsessive-compulsive disorder: is it time for the concept of an “autoimmune OCD” subtype?. Transl Psychiatry.

[150] Endres D (2022). Cerebrospinal fluid biomarkers for the detection of autoimmune depression. Biol Psychiatry.

[151] Zandi MS (2015). Clinical relevance of serum antibodies to extracellular N-methyl-D-aspartate receptor epitopes. J Neurol Neurosurg Psychiatry.

[152] Hansen N (2025). Neural autoantibodies in psychiatric disorders are associated with antibodies against viral pathogens: a retrospective study of 619 patients. J Neural Transm (Vienna).

[153] Hansen N (2020). Autoantibody-associated psychiatric symptoms and syndromes in adults: A narrative review and proposed diagnostic approach. Brain Behav Immun Health.

[154] Papi C (2025). Assessing commercial tissue-based assays for autoimmune neurologic disorders (II): antibodies to surface antigens. Neurol Neuroimmunol Neuroinflamm.

[155] Banwell B (2023). Diagnosis of myelin oligodendrocyte glycoprotein antibody-associated disease: International MOGAD Panel proposed criteria. Lancet Neurol.

[156] Mannara F (2020). Allosteric modulation of NMDA receptors prevents the antibody effects of patients with anti-NMDAR encephalitis. Brain.

[157] Dalmau J, Graus F (2018). Antibody-mediated encephalitis. N Engl J Med.

[158] Arino H (2016). Anti-LGI1-associated cognitive impairment: Presentation and long-term outcome. Neurology.

[159] Pollak TA, Moran N (2017). Emergence of new-onset psychotic disorder following recovery from LGI1 antibody-associated limbic encephalitis. BMJ Case Rep.

[160] Lai M (2009). AMPA receptor antibodies in limbic encephalitis alter synaptic receptor location. Ann Neurol.

[161] Hoftberger R A (2015). Encephalitis and AMPA receptor antibodies: Novel findings in a case series of 22 patients. Neurology.

[162] Graus F (2010). The expanding clinical profile of anti-AMPA receptor encephalitis. Neurology.

[163] Joubert B (2015). Clinical spectrum of encephalitis associated with antibodies against the α-Amino-3-Hydroxy-5-Methyl-4-Isoxazolepropionic acid receptor: case series and review of the literature. JAMA Neurol.

[164] Lancaster E (2010). Antibodies to the GABA(B) receptor in limbic encephalitis with seizures: case series and characterisation of the antigen. Lancet Neurol.

[165] Hoftberger R (2013). Encephalitis and GABAB receptor antibodies: novel findings in a new case series of 20 patients. Neurology.

[166] Jeffery OJ (2013). GABAB receptor autoantibody frequency in service serologic evaluation. Neurology.

[167] van Sonderen A (2016). The clinical spectrum of Caspr2 antibody-associated disease. Neurology.

[168] Irani SR (2012). Morvan syndrome: clinical and serological observations in 29 cases. Ann Neurol.

[169] Petit-Pedrol M (2014). Encephalitis with refractory seizures, status epilepticus, and antibodies to the GABAA receptor: a case series, characterisation of the antigen, and analysis of the effects of antibodies. Lancet Neurol.

[170] Pettingill P (2015). Antibodies to GABAA receptor α1 and γ2 subunits: clinical and serologic characterization. Neurology.

[171] Boronat A (2013). Encephalitis and antibodies to dipeptidyl-peptidase-like protein-6, a subunit of Kv4.2 potassium channels. Ann Neurol.

[172] Tobin WO (2014). DPPX potassium channel antibody: frequency, clinical accompaniments, and outcomes in 20 patients. Neurology.

[173] Hara M (2017). DPPX antibody-associated encephalitis: Main syndrome and antibody effects. Neurology.

[174] Spatola M (2018). Encephalitis with mGluR5 antibodies: Symptoms and antibody effects. Neurology.

[175] Munoz-Lopetegi A (2020). Sleep disorders in autoimmune encephalitis. Lancet Neurol.

[176] Tellez-Martinez A (2023). Suicidal thoughts and behaviors in anti-NMDA receptor encephalitis: psychopathological features and clinical outcomes. J Neuropsychiatry Clin Neurosci.

[177] Florance NR (2009). Anti-N-methyl-D-aspartate receptor (NMDAR) encephalitis in children and adolescents. Ann Neurol.

[178] Gibson LL (2019). The psychiatric phenotype of anti-NMDA receptor encephalitis. J Neuropsychiatry Clin Neurosci.

[179] Ramirez-Bermudez J (2024). Examining the features of neuroleptic malignant syndrome in anti-NMDA receptor encephalitis: a case-control study. J Acad Consult Liaison Psychiatry.

[180] Endres D (2021). An observational study on the association of anti-thyroid autoantibodies with clinical, EEG, MRI, FDG-PET, cerebrospinal fluid and anti-neuronal antibody findings in 530 patients with schizophreniform and affective disorders. Psychoneuroendocrinology.

[181] Hoffmann C (2021). The search for an autoimmune origin of psychotic disorders: Prevalence of autoantibodies against hippocampus antigens, glutamic acid decarboxylase and nuclear antigens. Schizophr Res.

[182] Arino H (2021). Real-world experience of assessing antibodies against the N-methyl-D-aspartate receptor (NMDAR-IgG) in psychiatric patients. A retrospective single-centre study. Brain Behav Immun.

[183] Soltani M (2022). A study of autoantibodies against some central nervous system antigens and the IL-35 serum level in schizophrenia. Iran J Allergy Asthma Immunol.

[184] Endres D (2022). Spectrum of novel anti-central nervous system autoantibodies in the cerebrospinal fluid of 119 patients with schizophreniform and affective disorders. Biol Psychiatry.

[185] Zhou D (2022). Rare presence of autoantibodies targeting to NMDA and GABA_A_ receptors in schizophrenia patients. Schizophr Res.

[186] Maier HB (2024). The significance of cerebrospinal fluid analysis in the differential diagnosis of 564 psychiatric patients: multiple sclerosis is more common than autoimmune-encephalitis. Psychiatry Res.

[187] Luykx JJ (2024). Clinical symptoms and psychosocial functioning in patients with schizophrenia spectrum disorders testing seropositive for anti-NMDAR antibodies: a case-control comparison with patients testing negative. Lancet Psychiatry.

[188] Enokida T (2024). Neuronal autoantibodies in the cerebrospinal fluid of 148 patients with schizophrenia and 151 healthy controls. Heliyon.

[189] Saito T (2020). An exploratory investigation of antibodies to NMDA-type glutamate receptor subunits in serum and cerebrospinal fluid among psychiatric patients with anti-thyroid antibodies. Heliyon.

[190] He J (2024). Multiple serum anti-glutamate receptor antibody levels in clozapine-treated/naïve patients with treatment-resistant schizophrenia. BMC Psychiatry.

[191] Bien CG (2021). Neural autoantibodies in cerebrospinal fluid and serum in clinical high risk for psychosis, first-episode psychosis, and healthy volunteers. Front Psychiatry.

[192] Theorell J (2021). Screening for pathogenic neuronal autoantibodies in serum and CSF of patients with first-episode psychosis. Transl Psychiatry.

[193] Jeppesen R (2023). Antineuronal antibodies in cerebrospinal fluid and serum of 104 patients with psychotic disorders compared to 104 individually matched healthy controls. Schizophr Res.

[194] Paval D (2024). Neural antibodies in first-episode psychosis patients with warning signs for autoimmune encephalitis. Clin Psychopharmacol Neurosci.

[195] Treiman G (2024). Rate of autoimmune encephalitis in children with first-episode psychosis. Pediatr Neurol.

[196] Tong J (2019). Elevated serum anti-NMDA receptor antibody levels in first-episode patients with schizophrenia. Brain Behav Immun.

[197] Loureiro CM (2021). Plasma prevalence of anti-N-methyl-d-aspartate receptor IgG antibodies in early stages of psychosis. Cien Saude Colet.

[198] Chan F (2021). Voltage-gated potassium channel blanket testing in first-episode psychosis: Diagnostic nihilism?. Aust N Z J Psychiatry.

